# Maternal Xp22.31 copy-number variations detected in non-invasive prenatal screening effectively guide the prenatal diagnosis of X-linked ichthyosis

**DOI:** 10.3389/fgene.2022.934952

**Published:** 2022-08-31

**Authors:** Xinxin Tang, Zhiwei Wang, Shuting Yang, Min Chen, Yue Zhang, Fang Zhang, Juan Tan, Ting Yin, Leilei Wang

**Affiliations:** Department of Prenatal Diagnosis, Lianyungang Maternal and Child Health Hospital, Lianyungang, China

**Keywords:** maternal CNV, *STS* gene, non-invasive prenatal screening, Xp22.31 microdeletion, prenatal diagnosis, X-linked ichthyosis

## Abstract

**Background and aims:** X-linked ichthyosis (XLI) is a common recessive genetic disease caused by the deletion of *steroid sulfatase (STS)* in Xp22.31. Maternal copy-number deletions in Xp22.31 (covering *STS*) can be considered an incidental benefit of genome-wide cell-free DNA profiling. Here, we explored the accuracy and clinical value of maternal deletions in Xp22.31 during non-invasive prenatal screening (NIPS).

**Materials and methods:** We evaluated 13,156 pregnant women who completed NIPS. The maternal deletions in Xp22.31 revealed by NIPS were confirmed with maternal white blood cells by chromosome microarray analysis (CMA) or copy-number variation sequencing (CNV-seq). Suspected positive women pregnant with male fetuses were informed and provided with prenatal genetic counseling.

**Results:** Nineteen maternal deletions in Xp22.31 covering *STS* were detected by NIPS, which were all confirmed, ranging in size from 0.61 to 1.77 Mb. Among them, eleven women with deletions in male fetuses accepted prenatal diagnoses, and finally nine cases of XLI were diagnosed. The nine XLI males had differing degrees of skin abnormalities, and of them, some male members of ten families had symptoms associated with XLI.

**Conclusion:** NIPS has the potential to detect clinically significant maternal X chromosomal CNVs causing XLI, which can guide the prenatal diagnosis of X-linked ichthyosis and reflect the family history, so as to enhance pregnancy management as well as children and family members’ health management.

## Introduction

Non-invasive prenatal screening (NIPS) based on analysis of cell-free DNA (cfDNA) in maternal peripheral blood plasma was developed to detect fetal aneuploidies, mainly targeting trisomies 21, 13, and 18. Because of its higher accuracy and sensitivity than those of traditional serological screening, NIPS has been used for routine prenatal screening on a large scale (van der Meij et al., 2019; Badeau et al., 2015). Notably, with the accumulation of clinical experience and the advancement of sequencing technology, NIPS based on genome massive parallel sequencing allows us to find the aforementioned viable trisomies, as well as other fetal aneuploidies, and even has the ability to detect subchromosomal copy-number variations (CNVs) in the fetal genome (Ye et al., 2021; Advani et al., 2017). A genome-wide cfDNA analysis has also enabled the detection of maternal CNVs with high resolution as nearly 90% of cfDNA in the maternal circulation originates from maternal cells. Therefore, NIPS has the potential to detect inherited maternal CNVs that could potentially be harmful for the fetus (Bianchi et al., 2015; Brison et al., 2017).

Recessive X-linked ichthyosis (XLI; OMIM: 308100) is an inherited dermatological condition characterized by generalized dryness and scaling of the skin, in which the preauricular area, neck, axillae, abdomen, and extension zone of the limbs are affected (Traupe et al., 2014). Additionally, some medical comorbidities have been identified in XLI cases, such as corneal deposits in the Descemet membrane, cryptorchidism, and increased risk of cardiac arrhythmias (Brcic et al., 2020). XLI is etiologically due to a deficiency of *steroid sulfatase (STS)* gene (OMIM: 300747) located on chromosome Xp22.31, 7 Mb from the p-telomere. Nearly 85∼90% of XLI cases are caused by ∼2 Mb microdeletion of the entire *STS* gene and flanking sequences, with the remaining cases attributable to *STS* point mutations or partial deletions. It almost exclusively affects males as it is an X-linked recessive genetic disease, with an incidence of 1/6,000 ∼ 1/2,000 in males upon clinical samples (Canueto et al., 2010; Wang et al., 2018) while approximately one in 1,500 upon prenatal screening data (Craig et al., 2010). Moreover, Craig et al. (2010) reported that the XLI prevalence of males in Asians (1/1,790) was comparable to Hispanics (1/1,620), while it was a little higher among non-Hispanic Whites (1/1,230), but the difference was not significant (Craig et al., 2010). Given that a large proportion of variants in the *STS* gene are inherited CNVs, NIPS based on genome-wide sequencing is expected to detect carrier mothers for XLI and guide the prenatal diagnosis according to the sex of the fetus.

Growing evidence from clinical studies has shown that NIPS can detect different types of maternal chromosomal aberrations such as unbalanced translocation, segmental autosomal abnormalities, or sex chromosome copy-number abnormalities (Wang et al., 2021). Increasing alertness to chromosomal abnormalities in the mother, which if inherited, could potentially be harmful to the fetus, is beneficial in promoting the health of the fetus and/or the mother. Brison et al. (2017) analyzed five maternal incidental findings during NIPS and proposed the reporting of those variants if clinically relevant. In 2019, Brison et al. (2019) analyzed the data of maternal CNVs detected in the *DMD* gene revealed by NIPS and proposed that NIPS results can return relevant and clinically actionable variants to the mother. However, current expertise regarding the accuracy in detecting the size and location of maternal CNV in Xp22.31 in NIPS and the guiding role of prenatal diagnosis of XLI are both limited. Research on the clinical benefits of the identification of XLI by NIPS and the ensuing dilemmas has hardly been reported. In this study, we analyzed 19 cases of maternal CNVs involving the *STS* gene detected by NIPS and explored the clinical application value and sociological significance of NIPS in the screening of XLI.

## Materials and methods

### Ethics statement and patients

**FIGURE 1:**
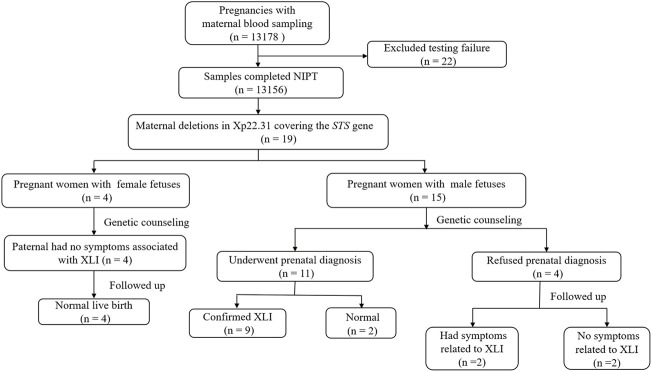
Flow diagram of the study. Abbreviations: NIPS, non-invasive prenatal screening; *STS*: *steroid sulphatase*; XLI: X-linked ichthyosis.

This study was approved by the Ethics Committee of Lianyungang Maternal and Child Health Hospital (Number: LYG-ME202007), and written informed consent was obtained from all patients before screening. A total of 13,178 pregnant women were referred for NIPS between January 2019 and December 2020 at the Prenatal Diagnosis Centre of Lianyungang Maternal and Child Health Hospital. The pregnant women had singleton pregnancies, and the maternal age as well as gestational age ranged from 19 to 47 years (mean age, 30.8 years) and 12^+1^ to 27^+6^ weeks (mean gestational age, 17.9 weeks), respectively. Clinical counseling was provided for all participants before NIPS. A detailed flow diagram of the study design is shown in Figure 1.

### Non-invasive prenatal screening

Rational NIPS and CNV detection were carried out as previously described (Chen et al., 2013; Pei et al., 2020). Briefly, a whole blood sample (5 ml) was collected from pregnant women and placed in tubes with EDTA. Plasma was separated within 8 h after blood collection. cfDNA extraction, sequencing library construction, and DNA nanoball (DNB) preparation were performed according to the standard operating procedures (BGI). Then, DNBs were sequenced on a BGISEQ-500 sequencing platform (BGI, Shenzhen, China) with the combined probe anchored polymer sequencing method. All sequencing data analyses were performed on original BGI software, Halos-NIFTY. The quality control parameters were as follows: the library concentration ranged from 5 to 50 ng/μL; effective data quantity was higher than 3.5 Mb; the GC content was 38%–42%; and the fetal DNA fraction was higher than 3.5%. The Z-scores for each chromosome were calculated, and the normal range for Chr21, Chr18, and Chr13 were defined as −3 < Z < 3 to identify fetal trisomies. The FCAPS algorithm was used for CNV identification as previously described by Chen et al. (2013), who employed a regression-based GC correction strategy, binary segmentation for breakpoint localization, and dynamic threshold for signal filtering. The location and size of CNV as well as the source of CNV were determined by binary segmentation and Z-test combined with fetal concentration. (Srinivasan et al., 2013). If the Z-score of the CNV in Xp22.31 is <-5, it will be defined as maternal CNV.

### Copy-number variation sequencing

Maternal genomic DNA was extracted from maternal white blood cells. A commercial CNV detection kit (BGI) was used to conduct whole-genome sequencing of nucleic acids for CNV detection on the MGISEQ-2000 sequencing platform. The sequencing type was SE45 (single-end sequencing, read length 45 bp), and the average sequencing depth was 0.4×. GRCh37 was used as the human genome reference sequence (UCSC database, http://genome.ucsc.edu/cgibin/hgGateway).

### Chromosome microarray analysis

The chromosome microarray analysis of fetal amniotic fluid cells was performed according to the procedure described in our previous study (Wang et al., 2019). DNA was extracted using a QIAamp DNA Mini Kit (Qiagen Inc. Valencia, CA, United States). After amplification, labeling, and hybridization with fragmented DNA, Affymetrix CytoScan 750 K (CytoScan 750 K Array; Affymetrix) chips were used for SNP array tests. The data were analyzed using the Chromosome Analysis Suite v3.2 (ChAs) software package (Affymetrix).

### Bioinformatics analysis for copy-number variations

For the interpretation of the data, the following public databases were used: Database of Genomic Variants (DGV; http://projects.tcag.ca/variation/), Database of Chromosomal Imbalance and Phenotype in Humans Using Ensembl Resources (DECIPHER; http://decipher.sanger.ac.uk/), Online Mendelian Inheritance in Man (OMIM; http://www.omim.org, ISCA search (http://dbsearch.clinicalgenome.org/search/), and NCBI ClinVar (https://www.ncbi.nlm.nih.gov/clinvar/). Pathogenicity analysis of CNVs was performed according to the standards and guidelines of the American College of Medical Genetics and Genomics (ACMG) (Riggs et al., 2020).

### Follow-up

Prenatal serological screening results, family history information, pregnancy outcomes, and neonatal physical manifestations were collected by consulting the local health information system as well as conducting telephone interviews. Follow-ups were performed up to at least 3 months after childbirth.

## Results

### Non-invasive prenatal screening for maternal copy-number variations in Xp22.31

Excluding the 22 pregnant women who failed to yield sequence results due to the low fetal fraction of cfDNA in NIPS, 13,156 reliable results were finally obtained. Of them, 27.5% women were advanced-maternal-age (AMA, age ≥35 years), 16.5% were at high risk of serological screening, 38.85% were at intermediate risk of serological screening, and the remaining pregnant women opted voluntarily for NIPS without any fingers mentioned previously. The average fetal fraction was 9.45%, ranging from 3.5% to 41.58%, and the average sequence unique reads was 11.15 Mb, ranging from 3.66 to 30.62 Mb. Among them, 19 maternal microdeletions were detected in Xp22.31 (covering the *STS* gene), among which 15 cases were revealed as pregnant with male fetuses and four cases with female fetuses by NIPS. The size of the aforementioned 19 CNVs ranged from 0.81 Mb to 1.98 Mb (mean size, 1.52 Mb). This corresponds to an incidence of 1/692 pregnant women who underwent NIPS.

### Maternal leukocyte verification by chromosome microarray analysis or copy-number variation sequencing

To confirm the respective maternal copy-number deletions in Xp22.31 detected by NIPS, DNA was extracted from maternal white blood cells, and chromosome microarray analysis (CMA) (cases 13, 14, and 17) and CNV-seq (the remaining cases) were conducted. The size and position of the 19 copy-number deletions of Xp22.31 detected by NIPS were confirmed, ranging in size from 0.61 to 1.77 Mb (mean size, 1.60 Mb). The data are summarized in Table 1.

**TABLE 1:** Size and position of the CNVs of Xp22.31: validation of chromosomal positions and size predicted by NIPS using CMA or CNV-seq.

Case	NIPS	Size (Mb)	Maternal white blood cell sequencing results	Size (Mb)	Method
1	del (X:6439315–7560506)-M	1.12	Xp22.31 (6420555–8147809)x1	1.73	CNV-Seq
2	del (X:6439315–8319869)-M	1.88	Xp22.31 (6433077–8192710)x1	1.76	CNV-Seq
3	del (X:6338138–8219054)-M	1.88	Xp22.31 (6433077–8153336)x1	1.72	CNV-Seq
4	del (X:6439315–7560506)-M	1.12	Xp22.31 (6427993–8153336)x1	1.73	CNV-Seq
5	del (X:6237892–8219054)-M	1.98	Xp22.31 (6494545–7989784)x1	1.50	CNV-Seq
6	del (X:6439315–7560506)-M	1.12	Xp22.31 (6433077–8147809)x1	1.71	CNV-Seq
7	del (X:7711037–8525366)-M	0.81	Xp22.31 (7819387–8427710)x1	0.61	CNV-Seq
8	del (X:6439315–8319869)-M	1.88	Xp22.31 (6427993–8147809)x1	1.72	CNV-Seq
9	del (X:6439315–8219054)-M	1.78	Xp22.31 (6378583–8147809)x1	1.77	CNV-Seq
10	del (X:6439315–7560506)-M	1.12	Xp22.31 (6459022–7841223)x1	1.38	CNV-Seq
11	del (X:6338138–8219054)-M	1.88	Xp22.31 (6427993–8153336)x1	1.73	CNV-Seq
12	del (X:6439315–7560506)-M	1.12	Xp22.31 (6459022–7950672)x1	1.49	CNV-Seq
13	del (X:6439315–8219054)-M	1.78	Xp22.31 (6455151–8143509)x1	1.69	CMA
14	del (X:6439315–8219054)-M	1.78	Xp22.31 (6455151–8135568)x1	1.68	CMA
15	del (X:6439315–8219054)-M	1.78	Xp22.31 (6420555–8159374)x1	1.74	CNV-Seq
16	del (X:6338138–7560506)-M	1.22	Xp22.31 (6427993–8153336)x1	1.73	CNV-Seq
17	del (X:6439315–8219054)-M	1.78	Xp22.31 (6449836–8135568)x1	1.69	CMA
18	del (X:6439315–7560506)-M	1.12	Xp22.31 (6573928–7878957)x1	1.31	CNV-Seq
19	del (X:6439315–8219054)-M	1.78	Xp22.31 (6393988–8147809)x1	1.75	CNV-Seq

### Prenatal diagnosis by chromosome microarray analysis

**FIGURE 2:**
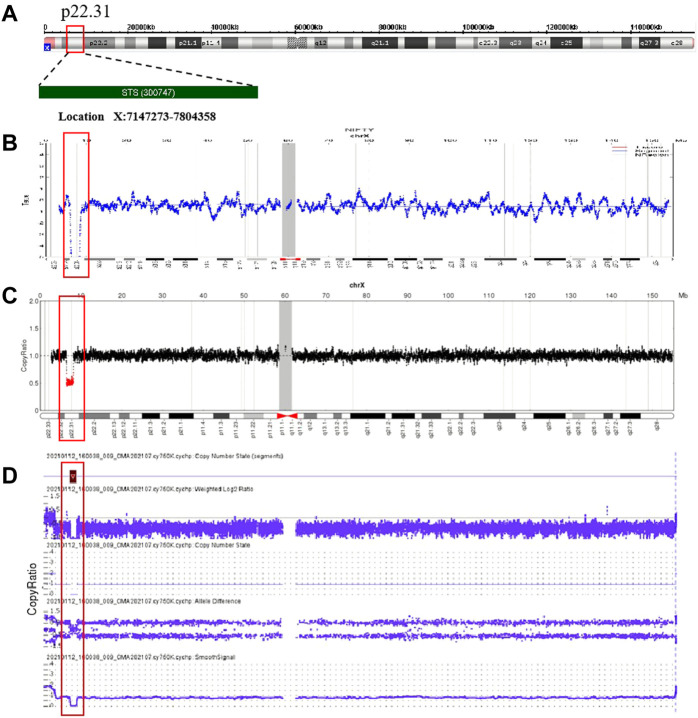
Non-invasive prenatal screening (NIPS), CNV-sequencing (CNV-seq), and CMA data plots for case 19. **(A)** Ideogram of chromosome X highlighting the Xp22.31 region encompassing the *STS* gene. **(B)** NIPS analysis of the maternal sex chromosome CNV plots: 46, XY, del(X) (p22.31) (1.78 Mb); **(C)** CNV-seq analysis of the sex chromosomes in maternal genomic DNA: 46, XY, del(X) (p22.31) (1.75 Mb); **(D)** CMA of the fetal amniotic fluid cells: 46, XY, del(X) (p22.31) (1.69 Mb).

After the 19 deletions in maternal CNVs of Xp22.31 detected by NIPS were confirmed by maternal white blood cells, prenatal genetic counseling was recommended, and amniocentesis for CMA was to be chosen voluntarily. The aforementioned 19 pregnant women were informed and were advised to undergo genetic counseling. After genetic counseling, the fathers of the four female fetuses had no symptoms associated with XLI, and the pregnant women were advised to continue the pregnancy. Eleven out of fifteen women with male fetuses underwent prenatal diagnosis with CMA testing (rate of prenatal diagnosis, 57.89%). The results of amniotic fluid CMA showed that copy-number deletions of Xp22.31 were observed in nine cases (cases 9, 11–15, and 17–19) without other chromosomal abnormalities, and the remaining two cases (cases 10 and 16) showed no noticeable abnormalities. The results of analysis using the OMIM database showed that case 11 had a deletion of five OMIM genes (*PUDP*, *STS*, *VCX*, *PNPLA4*, and *VCX2*), and the remaining cases had a deletion of four OMIM genes (*PUDP*, *STS*, *VCX*, and *PNPLA4*) without larger deletions involving contiguous genes detected. Four cases (case 5-8) refused prenatal diagnosis after genetic counseling. Figure 2 shows an example (case 19) of maternal copy-number deletions in Xp22.31 detected by NIPS, CNV-seq result of maternal white blood cells, and CMA result of amniotic fluid cells.

### Results of follow-up

Four women (case 1–4) with female fetuses and two women (cases 10 and 16) with no obvious abnormalities results of amniocentesis were delivered smoothly, and the babies had no symptoms associated with ichthyosis. Four women (cases 5–8) refused to conduct a diagnosis. Of them, two babies (cases 6 and 8) had symptoms related to XLI such as dry and scaly skin, while the remaining two (cases 5 and 7) had no relevant symptoms. Among the eleven women (case 10–19) who underwent prenatal diagnosis with CMA, the nine confirmed XLI males had different degrees of skin abnormalities. Some male relatives of the ten families (cases 4, 6, 8–9, 12–15, 18, and 19) may have XLI as their skin shows symptoms of the disease. The detailed clinical manifestations of the 19 cases with maternal deletion of CNVs causing XLI and family history are shown in Table 2.

**TABLE 2:** Results of prenatal diagnosis and follow-up of 19 deletion carriers.

Case	Sex	SNP-array	Deleted *OMIM gene*	Phenotype	Age	Family history
1	Female	No diagnosis	-	N	3 months	Not determined
2	Female	No diagnosis	-	N	3 months	Not determined
3	Female	No diagnosis	-	N	3 months	Not determined
4	Female	No diagnosis	-	N	3 months	Pregnant woman’s male cousin had abnormal skin resembling that of XLI
5	Male	No diagnosis	-	N	3 months	Not determined
6	Male	No diagnosis	-	Dryness and the light brown scales adherent to waist, abdomen and extension zone of the limbs, and dandruff	24 months	Pregnant woman’s father had abnormal skin resembling that of XLI
7	Male	No diagnosis	-	N	2 months	Not determined
8	Male	No diagnosis	-	Dryness and the white scales adherent to the legs and ankles and dandruff	24 months	Pregnant woman’s father had abnormal skin resembling that of XLI
9	Male	arr [hg19] Xp22.31 (6,455,151–8,135,568)x0	PUDP, STS, VCX, and PNPLA4	Dryness and the white scales adherent to the waist, legs, and abdomen	19 months	Pregnant woman’s brother had abnormal skin resembling that of XLI
10	Male	N	-	N	3 months	Not determined
11	Male	arr [hg19] Xp22.31 (6,455,151–8,141,076)x0	PUDP, STS, VCX, PNPLA4, and VCX2	Dryness	19 months	Not determined
12	Male	arr [hg19] Xp22.31 (6,475,263–7,711,675)x0	PUDP, STS, VCX, and PNPLA4	Dryness and the white scales adherent to the waist, legs, and abdomen	19 months	Pregnant woman’s nephew (son of sister) and brother had abnormal skin resembling that of XLI
13	Male	arr [hg19] Xp22.31 (6,455,151–8,135,568)x0	PUDP, STS, VCX, and PNPLA4	Severe dryness and the white scales adherent to the abdomen and extension zone of the limbs	17 months	Pregnant woman’s nephew (son of sister), brother, and uncle (son of grandparents) had abnormal skin resembling that of XLI
14	Male	arr [hg19] Xp22.31 (6,455,151–8,135,568)x0	PUDP, STS, VCX, and PNPLA4	Slight dryness and scales on instep skin	6 months	Pregnant woman’s male cousin had abnormal skin resembling that of XLI
15	Male	arr [hg19] Xp22.31 (6,455,151–8,135,568)x0	PUDP, STS, VCX, and PNPLA4	Dandruff	12 months	Pregnant woman’s father had abnormal skin resembling that of XLI
16	Male	N	-	N	3 months	Not determined
17	Male	arr [hg19] Xp22.31 (6,455,151–8,135,568)x0	PUDP, STS, VCX, and PNPLA4	Dryness and the white scales adherent to the abdomen and extension zone of the limbs	11 months	Not determined
18	Male	arr [hg19] Xp22.31 (6,455,151–8,135,568)x0	PUDP, STS, VCX, and PNPLA4	Eczema on ankles and waist	5 months	Pregnant woman’s father had abnormal skin resembling that of XLI
19	Male	arr [hg19] Xp22.31 (6,449,836–8,135,568)x0	PUDP, STS, VCX, and PNPLA4	Dryness and the white scales adherent to the abdomen and extension zone of the limbs	5 months	Pregnant woman’ son and brother had abnormal skin resembling that of XLI

N: no obvious abnormalities.

We obtained the serological screening results of seven pregnant women, shown in Table 3. The serum free estriol levels of the six pregnant women (cases 11–13, 17–19) with *STS* deficiency fetuses were all extremely low, with the minimum and maximum multiples of the median being 0.02 and 0.1, respectively, which were much lower than the reference range (0.7–2.5 multiples of the median), while the serum free estriol of pregnant women carrying normal fetuses (case 16) was in the normal range (0.74).

**TABLE 3:** Serum free estriol in the serum of the pregnant women with definite prenatal diagnosis.

Case	Gestational age	SNP-array of fetal amniotic fluid cells	Maternal serum screening		
			Value risk	uE3 (nmol/L)	uE3 MOM
11	17 + 5	arr [hg19] Xp22.31 (6,455,151–8,141,076)x0	DS:1/807;ES:1/111	0.657	0.1
12	16 + 2	arr [hg19] Xp22.31 (6,475,263–7,711,675)x0	DS:1/388;ES:1/56	0.229	0.05
13	17 + 1	arr [hg19] Xp22.31 (6,455,151–8,135,568)x0	DS:1/1713;ES:1/99	0.133	0.02
16	16 + 4	N	DS:1/606;ES:1/100000	3.41	0.74
17	17 + 2	arr [hg19] Xp22.31 (6,455,151–8,135,568)x0	DS1:1/2586;ES:1/677	0.166	0.03
18	16 + 4	arr [hg19] Xp22.31 (6,455,151–8,135,568)x0	DS:1/198;ES:1/5673	0.132	0.03
19	17 + 3	arr [hg19] Xp22.31 (6,449,836–8,135,568)x0	DS:1/38;ES:1:2761	0.116	0.02

N: no obvious abnormalities; DS: Down syndrome; ES: Edwards’ syndrome; uE3: unconjugated estriol; MOM: multiple of the median.

## Discussion

In this study, we analyzed the results of 13,156 NIPS cases and found 19 maternal copy-number deletions in the region of Xp22.31 (covering the *STS* gene). Because the majority of NIPS population in our center is from increased-risk pregnant women, it does not represent the local population of reproductive women. Thus, the incidence of CNVs of Xp22.31 is 1/692 in pregnant women who underwent NIPS, which does not include all pregnant women. Brcic et al. (2020) reported that the prevalence of *STS* deletion in females was 1/730 in 40–69-year-old participants from the United Kingdom Biobank resource, which is comparable to this study although from different groups of people, while Cavenagh et al. (2019) indicated a little lower prevalence with approximately 1 in 1,200 females within the general population. The size of the 19 CNVs mentioned previously was confirmed to be 0.61–1.77 Mb (mean size, 1.60), which was comparable to previous reports (Brcic et al., 2020; Zhang et al., 2020; Bai Z et al., 2019).

Maternal plasma DNA sequencing has been widely used for screening common fetal trisomies (trisomies 21, 18, and 13), as well as sex chromosome aneuploidy (Gil et al., 2015). With the gradual promotion of its clinical application and the progress of sequencing technology, most studies focused on NIPS in fetal CNV and even monogenic inherited disease prenatal screening (Advani et al., 2017; Vermeulen et al., 2017). It was reported that NIPS has a high sensitivity for detecting fetal CNVs, especially for CNVs >10 Mb (Liu et al., 2016). cfDNA in the peripheral blood of pregnant women is derived from the placenta, maternal bone marrow, and fetus. Since NIPS is performed on DNA from this mixed sample, it can therefore reflect maternal as well as fetoplacental DNA (Bianchi, 2018). Because nearly 90% of the cfDNA in the maternal circulation comes from maternal cells, NIPS can theoretically detect CNVs in the maternal background with higher accuracy than that of fetus, and some studies have supported this suggestion (Bianchi, 2004; Lenaerts et al., 2019). Brison et al. (2017) reported that NIPS could detect the maternal origin of CNVs over 500 kb in length by SeqCBS analysis. In this study, all 19 maternal CNVs in Xp22.31 detected by NIPS were confirmed by CMA or CNV-seq. The size and position of the fragments are highly consistent with the NIPS prompt results, despite the smallest fragment being only 0.61 Mb in length. The detection capability of the BGI-500 platform with Halos analysis for maternal CNVs of Xp22.31 was much higher than that of fetal CNVs, which reminds us that NIPS has great clinical application in maternal CNVs of the chromosome.

**FIGURE 3:**
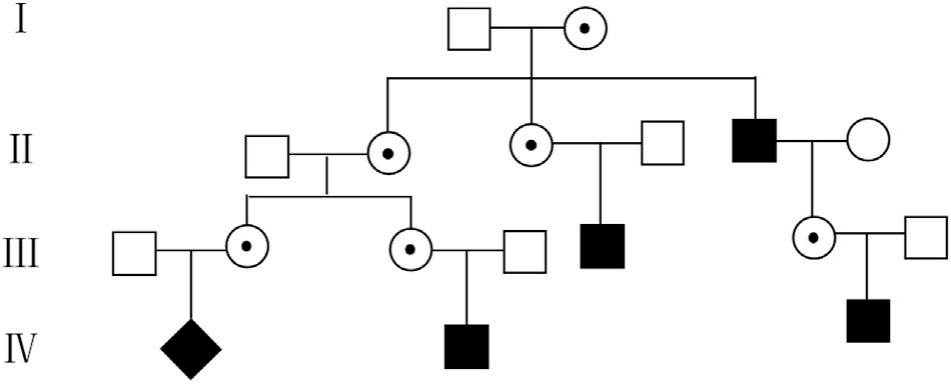
Pedigree 13 of the family with XLI according to the symptoms described by the pregnant woman. ◆, fetus with XLI; ■, suspicious affected male; 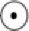, suspicious female carrier; □, healthy male; ○, healthy female.

Many scholars focus on the impact of maternal CNV on the discordant chromosome aneuploidies detected by NIPS. However, maternal CNV itself, in most cases, has important implications, thus interrogating that the maternal CNV landscape with clinical significance can help us improve overall pregnancy management (Brison et al., 2017). In this study, we detected 19 maternal copy-number deletions using NIPS on Xp22.31 involving the *STS* gene. Because XLI has the characteristics of X-linked recessive inheritance, if the mother is a carrier, half of the male offspring will be ichthyosis patients and half of the female offspring will be carriers. In addition, NIPS has the ability to indicate the sex of fetuses with high sensitivity and specificity (Bowman-Smart et al., 2020). Therefore, in this study, pregnant women with male fetuses were informed that the fetus had a 50% risk of XLI and were advised to undergo genetic counseling, while pregnant women with female fetuses were consulted regarding whether the father had symptoms associated with XLI. Finally, we found nine XLI male offspring by 13,156 NIPS results (1/1,461). Due to parents’ awareness of the disease during pregnancy, children with XLI received timely and effective treatment. In addition, Langlois et al. (2009) reported that 60% of patients had a positive family history. In this study, through communication with pregnant women, ten of them (52.6%) found that some male relatives of the family may have XLI because their skin showed symptoms of the disease. Taking pedigree 13 as an example, there were four men excluding the proband in the family who also showed dryness and brown or white scales attached to the skin. Still, regrettably, the four family members refused genetic testing. Thus, we can only speculate on the male and female carriers in the family based on the description of the pregnant woman (Figure 3). It was surprising that few knew about XLI and were not given proper treatment or genetic testing. Therefore, maternal copy-number deletions in the region of Xp22.31 detected by NIPS could also discover other carries in the family and help promote their disease management.

It has been reported that XLI with deletions of four OMIM genes (*PUDP*, *STS*, *VCX*, and *PNPLA4*) was characterized by mild-to-moderate abnormal skin appearance. Moreover, several complex clinical extracutaneous comorbidities such as corneal opacities, cryptorchidism, pyloric hypertrophy, bilateral periventricular nodular heterotopia, increased risk of cardiac arrhythmias, self-reported mood problems, heightened risk of psychopathology, and atypical seizures were also reported (Brcic et al., 2020; Trent et al., 2012; Fernandes et al., 2010; Puri et al., 2012). Here, the males of case 11 had deletions of five OMIM genes (*PUDP*, *STS*, *VCX*, *PNPLA4*, and *VCX2*), and the remaining cases had deletions of four OMIM genes (*PUDP*, *STS*, *VCX*, and *PNPLA4*) without larger deletions involving contiguous genes detected. Through follow-up, it was found that the nine XLI males had differing degrees of skin abnormalities. Cases 9, 12, 13, 17, and 19 had no skin abnormalities at birth, and 1 or 2 weeks after birth, the child’s skin appeared dry with white scales, mainly in the waist, abdomen, legs, and arms, and was aggravated in dry air but alleviated in a wet environment. However, cases 11, 14, and 15 showed light skin abnormalities. The male in case 15 was affected mainly in the scalp, and scratching of the scalp was inevitable due to itchiness, causing dandruff, while the rest of the body was unaffected. The males in cases 11 and 14 showed dryness with nothing serious. In addition, case 18 showed that the boy had severe eczema on his ankles and waist. Up to now, no aforementioned complex clinical extracutaneous comorbidities have been found. This may be due to the fact that the children we followed up are still young, and the oldest being only 24 months old. Thus, a more comprehensive clinical phenotype of XLI requires longer follow-ups.

As we know, XLI is caused by a deficiency of the *STS* gene. STS is a hydrolytic enzyme located in the endoplasmic reticulum, also known as arylsulfatase C. STS catalyzes the hydrolysis of the sulfate esters of 3-hydroxysteroids, such as dehydroepiandrosterone sulfate, cholesterol sulfate, androstenediol-3-sulfate, and pregnenolone sulfate, and produces biologically active steroids. Previous studies have demonstrated that a low level of serum free estriol in pregnant women was associated with STS deficiency due to a metabolic blockage (Jari et al., 2004; Piazzi et al.,. 1998; Diociaiuti et al., 2019). Langlois et al. (2009) reported that the uE3 levels of 30 pregnant women with prenatally diagnosed STS deficiency were all below 0.25 MoM. Coincidentally, Craig et al. (2010) reported that all the 74 pregnancies affected by XLI were associated with maternal serum uE3 values < 0.20 MoM. In this study, we obtained Down’s syndrome screening results in the second trimester for seven pregnancies (cases 11–13 and 16–19). Six pregnant women carrying XLI-affected male fetuses had high- or intermediate-risk screening results. The serum free estriol in the serum of the six pregnant women (11–13 and 17–19) were all extremely lower than the reference range (0.7–2.5 multiples of the median), while the serum free estriol of pregnant women carrying normal fetuses (case 16) was in the normal range (0.74). These results again corroborate previous reports. In addition, it is interesting to note that of the 15 pregnant women with maternal deletions in Xp22.31 with male fetuses, 11 (73.3%) of the males had confirmed or putative XLI, which was much higher than the theoretical 50%. This is probably because six of them had extremely low levels of uE3 values by serum screening, which makes the positive predictive value higher than 50%. This reminds us that maternal serum uE3 screening combined with NIPS has the potential to become a new and effective screening mode for XLI, but the application prospect still requires the accumulation of a large number of clinical data.

## Conclusion

In conclusion, NIPS has the potential to clinically detect significant maternal X chromosomal CNVs causing XLI, which can help to predict the probability of the fetus inheriting the mutation and guide the prenatal diagnosis of XLI, so as to enhance pregnancy management and child management. Moreover, through genetic counseling, it can also reflect the potential carriers of the family members so as to help family health management.

## Data Availability

The datasets for this article are not publicly available due to concerns regarding participant/patient anonymity. Requests to access the datasets should be directed to the corresponding author.
